# Web-Based Intervention for Family Carers of Persons with Dementia and Multiple Chronic Conditions (My Tools 4 Care): Pragmatic Randomized Controlled Trial

**DOI:** 10.2196/10484

**Published:** 2018-06-29

**Authors:** Wendy Duggleby, Jenny Ploeg, Carrie McAiney, Shelley Peacock, Kathryn Fisher, Sunita Ghosh, Maureen Markle-Reid, Jennifer Swindle, Allison Williams, Jean AC Triscott, Dorothy Forbes, Kathya Jovel Ruiz

**Affiliations:** ^1^ Faculty of Nursing University of Alberta Edmonton, AB Canada; ^2^ School of Nursing McMaster University Hamilton, ON Canada; ^3^ Department of Psychiatry and Behavioural Neurosciences McMaster University Hamilton, ON Canada; ^4^ College of Nursing University of Saskatchewan Saskatoon, SK Canada; ^5^ Department of Medical Oncology University of Alberta Edmonton, AB Canada; ^6^ Department of Mathematical and Statistical Sciences University of Alberta Edmonton, AB Canada; ^7^ School of Geography & Earth Sciences McMaster University Hamilton, ON Canada; ^8^ Department of Family Medicine Glenrose Rehabilitation Hospital Edmonton, AB Canada; ^9^ School of Nursing University of Western Ontario London, ON Canada

**Keywords:** carers, dementia, treatment, internet

## Abstract

**Background:**

My Tools 4 Care (MT4C) is a Web-based intervention that was developed based on the transitions theory. It is an interactive, self-administered, and portable toolkit containing six main sections intended to support carers of community-living persons with Alzheimer’s disease and related dementia and multiple chronic conditions through their transition experiences.

**Objective:**

The objective of our study was to evaluate the effectiveness of MT4C with respect to increasing hope, self-efficacy, and health-related quality of life in carers of community-living older persons with Alzheimer’s disease and related dementia and multiple chronic conditions.

**Methods:**

A multisite, pragmatic, mixed methods, longitudinal, repeated-measures, randomized controlled trial was conducted between June 2015 and April 2017. Eligible participants were randomized into either treatment (MT4C) or educational control groups. Following baseline measures, carers in the treatment group received 3 months of password-protected access to MT4C. Trained research assistants collected data from participants via phone on hope (Herth Hope Index [HHI]), self-efficacy (General Self-Efficacy Scale), and health-related quality of life (Short Form-12 item [version 2] health survey; SF-12v2) at baseline, 1, 3, and 6 months. The use and cost of health and social services (Health and Social Services Utilization Inventory) among participants were measured at baseline, 3, and 6 months. Analysis of covariance was used to identify group differences at 3 months, and generalized estimating equations were used to identify group differences over time.

**Results:**

A total of 199 carers participated in this study, with 101 participants in the treatment group and 98 in the educational control group. Of all, 23% (45/199) participants withdrew during the study for various reasons, including institutionalization or death of the person with dementia and lack of time from the carer. In the treatment group, 73% (74/101) carers used MT4C at least once over the 3-month period. No significant differences in the primary outcome measure (mental component summary score from the SF-12v2) by group or time were noted at 3 months; however, significant differences were evident for HHI-factor 2 (*P*=.01), with higher hope scores in the treatment group than in the control group. General estimating equations showed no statistically significant group differences in terms of mental component summary score at all time points. Attrition and the fact that not all carers in the treatment group used MT4C may explain the absence of statistically significant results for the main outcome variable.

**Conclusions:**

Despite no significant differences between groups in terms of the primary outcome variable (mental component score), the significant differences in terms of one of the hope factors suggest that MT4C had a positive influence on the lives of participants.

**Trial Registration:**

ClinicalTrials.gov NCT02428387; https://clinicaltrials.gov/ct2/show/NCT02428387 (Archived by Webcite at http://www.webcitation.org/708oFCR8h).

## Introduction

Web-based interventions have become increasingly popular as a means to support family and friend caregivers (hereafter carers) because of their flexibility and ease of access [1,2]. The importance of support for carers of persons with Alzheimer’s disease and related dementias (ADRD) has been acknowledged as a worldwide issue given the growing numbers of persons with ADRD and the recognition that the majority of their care is provided by carers [3]. The complexity of the care provided by carers is further complicated when the person with ADRD lives with the carer, in the community, and has multiple chronic conditions (MCC) [4]. As a result of caregiving, carers undergo significant life-altering transitions, such as changes in roles and relationships that can have a negative impact on their quality of life (QOL) [5]. Transitions are significant changes that individuals need to incorporate into their lives to obtain positive health outcomes [6]. The lack of resources to support carers through these transitions is compounded by the 24/7 nature of caregiving for persons with ADRD, which makes accessing resources difficult. Web-based interventions show promise because they can be accessed at times and places that are convenient for carers [2,7].

Three systematic reviews of Web-based interventions for carers of persons with ADRD [2,8,9] reported that interventions resulting in improved carer health outcomes, for example, reduced anxiety and stress, had the following features: (1) they could be individually tailored by incorporating choices in different parts of the intervention [10-12], (2) they offered multiple components [10,13,14], and (3) they were psychoeducational interventions [10,12,15-20]. Most of the reviewed studies were pilot studies with the authors recommending future research using pragmatic randomized controlled trial (RCT) designs to evaluate Web-based interventions. The most common theoretical foundations for the interventions were stress and burden theories, with a focus on strain and depression [21]. However, these theories do not address the multiple, complex, reoccurring, and significant life-altering changes and the processes of the changes (transitions) that carers experience throughout their caregiving experiences [21,22].

Based on an adaptation of Meleis’ theory of transition [6] and on our previous research that focused on transitions, hope, and QOL [5], a self-administered, Web-based intervention titled My Tools 4 Care (MT4C) [23] was developed in partnership with the Alzheimer’s Society of Alberta and Northwest Territories to support carers during their transition experiences. Transition experiences are the processes triggered by significant changes and involve carers acknowledging the changes, connecting with others, and redefining their perception of normal [22]. Redefining the perceptions of what is normal results in decreased stress and increased hope and makes one feel confident [24]. As part of the development of MT4C, Duggleby et al’s [22] adaptation of Meleis’ transition theory, involving core concepts of acknowledging the situation, connecting, and redefining normal, was mapped to the specific components of MT4C (for more information see Duggleby et al) [22].

The Web developers for this intervention were ATMIST [25]. A hard copy version of MT4C was pilot-tested by 20 carers of persons with ADRD, who found it feasible, acceptable, and potentially able to support them through transitions [22]. The principles that guided the development of the Web-based MT4C intervention were as follows: (1) inclusion of choice, the carers choose which sections they would like to use and when; (2) encouragement of user-generated content, carers can write in sections, add stories, pictures, music, etc; (3) portability, available on the Web, tablet, or mobile phone; and (4) privacy of information, only the carers can view their entries and share the contents.

The purpose of this pragmatic RCT was to evaluate the effectiveness of MT4C with respect to increasing health-related quality of life (HRQOL), self-efficacy, and hope among carers of older adults with ADRD and MCC in the community. Participants in the treatment group used MT4C for 3 months. We hypothesized that participants using MT4C would have increased hope, improved general self-efficacy, and increased HRQOL scores at 3 months compared with those at baseline and compared with those exhibited by an educational control group, at no additional cost. The following research questions were addressed in this study:

1. Does use of MT4C result in a 3-month (immediately post intervention) and 6-month (3 months post intervention) increase in HRQOL, self-efficacy, and hope in carers of persons with ADRD and MCC compared with that in an educational control group?
2. Are the effects or benefits of MT4C achieved at no additional cost in the treatment group compared with that in an educational control group?

## Methods

### Design

A detailed protocol for this study has been published elsewhere [26]; thus, only a summary has been provided here. This trial has been reported in accordance with the CONSORT-EHEALTH checklist [27]. The study design was a multisite, pragmatic, mixed-methods, longitudinal, repeated-measures, RCT. Baseline data were collected, followed by random assignment to a treatment (MT4C) or educational control group. Measures were repeated at 1, 3, and 6 months. As this was a mixed-methods concurrent study with a predominately quantitative design [28], qualitative data (semistructured interviews) were collected concurrently with, and were used to inform, the quantitative data. Quantitative and qualitative data were integrated at the results stage. Data quality was checked throughout the study by research assistants and at monthly meetings by the research team.

There were no changes to the content of MT4C, bug fixes, or unexpected events in association with MT4C use during this study. The study received ethical approval from the University of Alberta Health Research Ethics Board (# Pro0004872) and the Hamilton Integrated Research Ethics Board (#15-309).

### Recruitment and Participants

Participant recruitment occurred offline over a 2-year period in 2 Canadian provinces (Ontario and Alberta) using multiple strategies. Trained research assistants targeted local branches of the Alzheimer’s Society, in both provinces, and attended education groups for carers and shared study-related information, including its purpose and inclusion criteria. In addition, staff at the Alzheimer’s Society and coordinators at community-based carer support groups, geriatric outpatient or memory clinics, adult day programs, and senior support services were provided with recruitment materials (eg, brochures or postcards). Staff members from these groups approached potential participants and obtained their consent to be contacted by the research team. In Alberta, advertisements in local community newspapers requested that interested carers contact the research coordinator using a toll-free number or via email.

Research assistants contacted interested carers via phone to screen for eligibility and to schedule the first interview. Participants were considered eligible to participate in the study if they were above the age of 18 years and were providing physical, emotional, or financial care for a community-living care recipient aged 65 years or older who had ADRD and two or more chronic conditions. The participants were all English speaking and either a family or friend of the care recipient, with access to a computer and a valid email address. Exclusion criteria included non-English speaking carers and those caring for a family member or friend who was under the age of 65 years, who did not have ADRD and MCC, or who was not a community-living care recipient.

### Randomization

Allocation of participants into treatment and educational control groups was achieved using a 1:1 ratio. A biostatistician, not involved in recruitment, generated group allocations using stratified permuted block randomization. Random number sequences were fed into RedCap, a secure, password-protected, Web-based randomization service offered at the University of Alberta, which allocated clients to the two groups according to a random sequence.

### Blinding

Given the nature of the study, the research team was unblinded to group allocation. Recruitment materials referred to evaluating different strategies to help carers and did not mention MT4C. Furthermore, to prevent contamination, participants were asked to keep the information about what group they were in confidential. To prevent participants from identifying the group to which they were allocated, two different consent forms were created, one for each group. These were used to obtain verbal telephone informed consent from each study participant. The consent form also acknowledged the potential risks that participants might encounter during the study (see Multimedia Appendix 1). Immediately following the first interview, participants received an email from the research assistants containing information on the study along with copies of the consent form and data collection tools. Participants allocated to the treatment group were provided with password-protected, no-cost access to MT4C.

**Figure 1:**
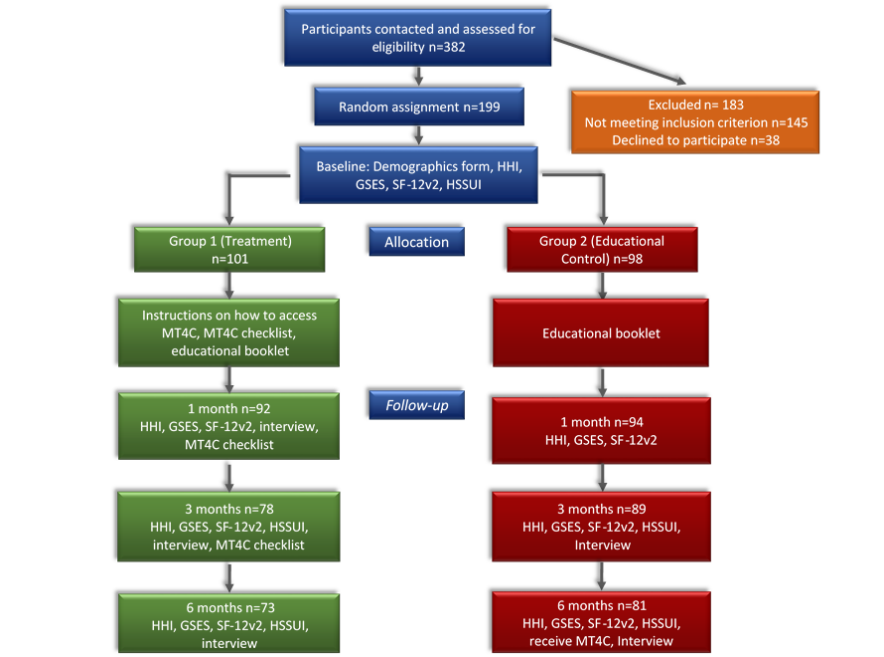
Number of Participants at Each Data Collection Period and Data Collection Procedure. GSES: General Self-Efficacy Scale; HHI: Herth Hope Index; HSSUI: Health and Social Services Utilization Inventory; MT4C: My Tools 4 Care; SF-12v2: Short Form-12 item health survey.

Data collection occurred from June 2015 to April 2017. Figure 1 outlines the data collection procedures, which are available in more detail in the study protocol article [24]. Trained research assistants, in each province, completed audiotaped telephone interviews (quantitative and qualitative) that lasted anywhere from 15 to 60 min.

### Intervention

Participants in the treatment group were instructed to access MT4C at their convenience on a computer, tablet, or mobile phone for 3 months. Once the carer logged on to the site, the first page (“How to use MT4C”) provided instructions on how to use MT4C and contained a menu outlining the sections constituting the toolkit. MT4C consists of six main sections: (1) about me, (2) common changes to expect, (3) frequently asked questions, (4) resources, (5) important health information, and (6) calendar. In the *About Me* section, participants have the option to add formatted text, pictures, and PDF files (see Multimedia Appendix 2 for screenshots of all MT4C pages). All data entered by participants into the site remained confidential, even from the study team. Participants also received an electronic copy of the Alzheimer’s Society’s *The Progression of Alzheimer’s Disease* booklet [29], a copy of the study questionnaires, and the MT4C toolkit checklist intended for participants to record their use of the MT4C site.

### Educational Control Group

Participants in the educational control group (usual care) received a copy of the Alzheimer’s Society’s *The Progression of Alzheimer’s Disease* booklet, via email, after the first interview. This booklet is freely available through the Alzheimer’s Society of Canada and provides information for the person with dementia, his or her family, and carers on the stages of Alzheimer’s disease. At the end of data collection, participants in the control group received an email containing a one-page summary of the study, including preliminary findings, and instructions on how to contact the research team if they wished to have access to the MT4C site.

### Measures

#### Demographics

All participants completed a demographic questionnaire during the baseline interview. Information collected included age, gender, marital status, ethnicity, citizenship, level of education, employment status, occupation, income, relationship to the person with ADRD, carer-specific chronic health conditions, and length of time spent caregiving. This form also collected information on the care recipients’ age, gender, and number of chronic conditions.

#### Toolkit Checklist

Participants accessing the MT4C site were asked to document, offline, the frequency and amount of time (in minutes) they spent on each section of the site. Research assistants reviewed this information with participants at the 1- and 3-month interviews. At the 1-month interview, participants were reminded that at 3 months, their access to MT4C would end, and they were encouraged to use the site, if not done so already, and continue completing the checklist.

#### Primary Outcome Measure: Short Form-12 Item (version 2) Health Survey

All participants completed the Short Form-12 item (version 2) health survey (SF-12v2) at all time points. It is a widely used measure of HRQOL consisting of 12 questions, measuring 8 domains of functioning and well-being (physical functioning, role functioning, bodily pain, general health, vitality, social functioning, emotional health, and mental health) [30,31]. Overall scores are summarized in 2 domains: a physical component summary score (PCS) and a mental component summary score (MCS). Scores range from 0 to 100, with higher scores indicating a better HRQOL. SF-12v2 is a reliable tool with estimated PCS and MCS test-retest reliabilities of *r*=0.89 and *r*=0.86, respectively [32]. MCS was selected as the primary outcome for this study, given the psychoeducational nature of the intervention.

#### Secondary Outcome Measures: SF-12v2 PCS, General Self-Efficacy Scale, and Herth Hope Index

Secondary outcome measures included the SF-12v2 PCS score (described above) and the General Self-Efficacy Scale (GSES) and Herth Hope Index (HHI) scores. Participants completed the GSES and HHI at all data collection time points. GSES was used to assess participants’ perceived self-efficacy or belief that they can complete novel or difficult tasks or cope with diversity [33]. It is a 10-item, 4-point scale with a Cronbach’s alpha coefficient of reliability r ranging from 0.76 to 0.90 (*P*<.05). Total scores range from 10 to 40, with higher scores indicating a greater level of self-efficacy. GSES has been used in countries around the world and has been adapted to 26 languages [33].

HHI is a 12-item Likert-type scale [34]. Items are scored from 1 “strongly disagree” to 4 “strongly agree.” Total scores range from 12 to 48, with higher scores indicating a greater hope. The items in the scale can be grouped into three factors of hope: (1) temporality and future, (2) positive readiness and expectancy, and (3) interconnectedness. HHI has been used in a variety of populations and has a test-retest reliability of 0.91 (*P*<.05) and criterion-related validity r of 0.81 to 0.92 (*P*<.05) [34].

#### Health and Social Services Utilization Inventory

A modified version of the Health and Social Services Utilization Inventory (HSSUI) was used to collect service use information to calculate costs from a societal perspective [35] from all study participants at baseline, 3 months, and 6 months. The initial version of HSSUI was developed by Browne and colleagues and has been shown to have good reliability and validity (*r*=0.72 to 0.99) [36,37]. This survey measures the use of services by asking respondents to think back over a specific period of time (here, 3 months) about the type of health and social services accessed, number of times used, and any out-of-pocket expenses related to these services. HSSUI was developed for persons with different types of illnesses. For our study with carers, it was modified to reflect service use such accessing the Alzheimer’s Society.

#### Qualitative Interviews

All participants were interviewed at 1, 3, and 6 months using semistructured, audiotaped telephone interviews. Questions were asked about the significant changes they had experienced as carers as well as what had helped them deal with these changes. At 3 and 6 months, participants using MT4C were asked about the following: (1) their perceptions of MT4C, (2) how MT4C helped them deal with transitions, (3) what they liked most and least about MT4C, and (4) changes they would make to MT4C.

### Data Analysis

#### Primary and Outcome Measures

All quantitative data were entered in SPSSv24 and cleaned and checked by a research assistant for accuracy. SAS version 9.4 was used for all statistical analyses. Statistical tests assumed a 0.05 two-tailed level of significance and 95% CIs.

Baseline characteristics data are presented as means and SDs for continuous variables and as numbers and percentages for categorical variables. Analysis of covariance (ANCOVA) was used to test the differences in outcome variables between the intervention and control groups at 3 months. The 3-month analysis represents the primary analysis for this study because this period corresponds to the duration of the intervention. Separate ANCOVA models were run for each outcome, with the 3-month outcome as the dependent variable, group (intervention, control) as the independent variable, and baseline value of the outcome as the covariate, rather than determining differences between groups at baseline (CONSORT guidelines) [38]. Intention-to-treat principles were used in all analyses; thus, all participant data were analyzed in the groups in which they were originally allocated (including those in the treatment group who did not utilize MT4C); imputation was applied to address missing data. Multiple imputation is considered the best method for addressing the most common and realistic missing data patterns seen in RCTs [39]. We performed multiple imputations using the general procedure and employing the fully conditional specification procedure with predictor mean matching [40]. A range of auxiliary and outcome variables were used in the imputation model to improve accuracy; thus, the imputation model included baseline variables (age and gender of carer, number of carer chronic conditions, and number of care recipient chronic conditions) as well as the secondary outcome variables. After missing data were imputed (five imputations), each dataset was analyzed using ANCOVA, and the results from these multiple analyses were pooled to obtain an overall inference. Sensitivity analyses were performed using the complete case dataset.

Subgroup analyses were performed to determine whether the differences in intervention effectiveness for the primary outcome were observed for specific baseline groups. These were restricted to the following six baseline factors: age, gender, carer employment status, number of carer chronic conditions, number of care recipient chronic conditions, and income. Subgroup differences in the intervention effect were determined based on the significance of the group or subgroup interaction term in the ANCOVA model.

Generalized estimating equations (GEE) were used to determine any group differences over the 6-month period. GEE was selected because it is a robust method that does not rely on normality assumptions to address the dependency in repeated-measures data [41]. This 6-month analysis is regarded as a secondary or supplemental analysis in this study because the 3- to 6-month period corresponds to a time when the intervention was no longer available to treatment group participants. Separate GEE models were run for each outcome (primary, secondary). GEE models included group (intervention, control), time, and group × time interaction; the group × time interaction was of primary interest because statistical significance for this variable indicates the presence of a treatment effect [41].

Cost analyses were conducted to compare the cost of health service use in the intervention and control groups. Neither the intervention nor control group had program-specific costs. The service use that clients reported, using HSSUI at baseline and at 3 months (end of intervention period), was multiplied by the unit costs for the service to obtain total service costs. Unit costs were obtained from Ontario and Alberta databases, which provide the costs of all services paid for by the publicly-funded health care systems in each province. Cost data are substantially positively skewed and have traditionally been handled using nonparametric methods [42]. Mann–Whitney U-test was used to evaluate the between-group differences in median costs and to compare the total service costs at baseline and 3 months between the two groups.

#### Qualitative Interview Data

Qualitative data were analyzed using a qualitative descriptive approach in which coding categories were derived directly from the data [43]. Each transcript was read as a whole looking for similarities, differences, and patterns. Categories were grouped into themes. Trustworthiness of the data was maintained using the words of participants as much as possible and keeping an audit trail of analytic decisions. The findings from the qualitative analysis were integrated with the quantitative findings; an in-depth analysis of the qualitative findings for this study is reported elsewhere [44].

## Results

### Participants

**Figure 2:**
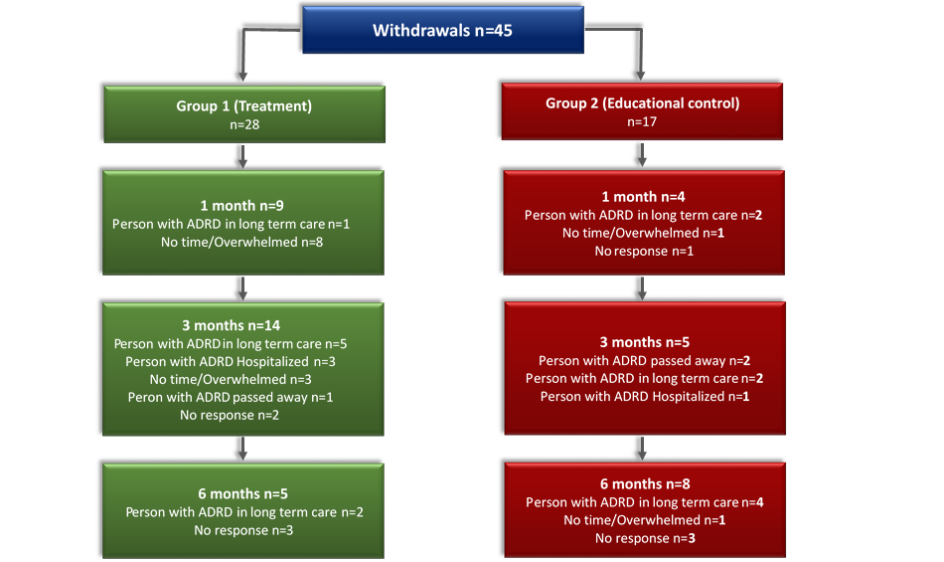
Number of Participant Withdrawals. ADRD: Alzheimer’s disease and related dementias.

Recruitment began on May 2015; a total of 382 persons were contacted, resulting in 199 carers of community-living persons with ADRD and MCC who participated in the study (Ontario: 106/199, 53.3% and Alberta: 93/199, 46.7%; Figure 1). Participants were randomly assigned to either treatment (n=101) or control (n=98) group. Of all participants, 22.6% (45/199) withdrew during the study (Figure 2), and a total of 154 (154/199, 77.4%) carers completed the study (treatment group: 73/154, 47.4%; control group, 81/154, 52.6%).

### Participants (Baseline Characteristics)

Table 1 shows participants’ baseline characteristics. Randomization resulted in no significant differences between the two groups. Most carers in the study (161/199, 80.9%) were female, married or in a common law relationship (168/199, 84.4%), Caucasian (185/199, 93.0%), unemployed (111/199, 55.8%), and either the spouse (98/199, 49.2%) or adult child (91/199, 45.7%) of the person with dementia. Of all carers, 69.3% (138/199) resided with the person with dementia and 68.8% (137/199) received assistance with caregiving. On average, carers in the study were 63.6 (SD 11.6) years of age and had been carers for approximately 4.3 (SD 4.2) years. Care recipients in the study were on average 80.3 (SD 7.7) years of age and had an average of 10.0 (SD 4.1) chronic conditions (Table 1).

### Intervention “Dose” (Use of My Tools 4 Care)

In the intervention group, 73% (74/101) carers used MT4C at least once over the 3-month period. At 1 month, participants who used the site spent the most time on Section 1 *My story* (median 9.5 min, interquartile range [IQR] 28.4). By 3 months, participants spent most of their time on Section 2 *Common changes to expect* (median 15 min, IQR 45.0) and Section 4 *Resources* (median 10.00 min, IQR 20.0).

### Intervention Effectiveness

The results of the multiple imputation ANCOVA testing for group differences in mean changes in the outcome scores from baseline to 3 months are provided in Table 2. No significant group differences were observed in the primary or secondary outcome measures. A significant group difference was observed for factor 2 of HHI, although no difference was observed for the overall HHI. The complete case ANCOVA results were consistent with the multiple imputation ANCOVA results (data not shown). Given the absence of effects at 3 months and termination of the intervention use at this time, GEE was expected to show no overall effect for the 6-month period.

**Table 1:** Demographic characteristics of caregivers and care recipients.

Characteristic				Intervention (n=101)		Control (n=98)		Total Sample (N=199)
**Carers**								
	Age, mean (SD)		63.4 (12.2)		63.9 (11.1)		63.6 (11.6)	
	Number of years caregiving, mean (SD)		4.1 (3.8)		4.6 (4.5)		4.3 (4.2)	
	Years of education, mean (SD)		14.1 (2.9)		14.27 (3.0)		14.18 (2.9)	
	Chronic conditions, mean (SD)		2.2 (1.5)		2.5 (1.6)		2.4 (1.6)	
	**Gender, n (%)**							
		Male	22 (22)		16 (16)		38 (19)	
		Female	79 (78)		82 (84)		161 (81)	
	**Marital status, n (%)**							
		Married or living with someone	84 (83)		85 (87)		168 (85)	
		Single, widowed, divorced or separated	17 (17)		13 (13)		30 (15)	
	**Ethnicity, n (%)**							
		Caucasian	93 (92)		92 (94)		185 (93)	
		Other	8 (8)		6 (6)		13 (7)	
	**Employed, n (%)**							
		Yes	39 (39)		47 (48)		86 (44)	
		No	61 (61)		50 (52)		111 (56)	
	**Relationship to care recipient, n (%)**							
		Spouse or life partner	51 (50)		47 (48)		98 (49)	
		Son or daughter	47 (47)		44 (45)		91 (46)	
		Other	3 (3)		7 (7)		10 (5)	
	**Living with care recipient, n (%)**							
		Yes	70 (69)		68 (69)		138 (69)	
		No	31 (30)		30 (31)		61 (31)	
	**Finances meet needs, n (%)**							
		Completely or Very well or Adequately	81 (80)		76 (79.2)		157 (80)	
		With some difficulty or Not very well or Totally inadequate	20 (20)		20 (20.8)		40 (20)	
	**Household income, n (%)**							
		Less than Can $40,000	25 (29)		24 (30.4)		49 (30)	
		Can $40,000 to $70,000	23 (27)		16 (20.3)		39 (23)	
		Greater than Can $70,000	38 (44)		39 (49.3)		77 (47)	
	**Assistance with caring, n (%)**							
		Yes	70 (69)		67 (68)		137 (69)	
		No	31 (31)		31 (32)		62 (31)	
**Care Recipient**								
	Age, mean (SD)		80.5 (7.4)		80.2 (8.0)		80.3 (7.7)	
	Chronic conditions, mean (SD)		10.4 (4.1)		9.6 (4.0)		10.0 (4.1)	
	**Gender, n (%)**							
		Male	55 (54)		49 (50)		104 (52)	
		Female	46 (46)		49 (50)		95 (48)	

**Table 2:** ANCOVA results using multiple imputation (baseline to 3 months).

Outcome Measures		Pooled LSM^a^ Group Difference (95% CI)	*P* value for Null Model (No Group Effect)
SF-12 v2 PCS^b^		−0.02 (−2.07 to 2.01)	.98
SF-12 v2 MCS^c^		−0.23 (−3.25 to 2.80)	.88
**HHI^d^**		0.56 (−0.25 to 1.36)	.17
	HHI-Factor 1	0.05 (−0.28 to 0.38)	.77
	HHI-Factor 2	0.56 (0.11 to 1.01)	.01^e^
	HHI-Factor 3	0.11 (−0.25 to 0.47)	.55
GSES^f^		0.22 (−0.78 to 1.22)	.67

^a^LSM:Least Square Means.

^b^SF-12 PCS: Short Form-12 item health survey physical component summary score.

^c^SF-12 MCS: Short Form-12 item health survey mental component summary score.

^d^HHI: Herth Hope Index.

^e^Significant at *P* ≤.05.

^f^GSES: General Self-Efficacy Scale.

**Table 3:** Generalized estimating equations with Short Form-12 item (version 2) mental component summary score as the dependent variable (all time points).

Parameter		Beta	SE	95% Wald CI	Hypothesis Test		
					Wald chi-square	*df* ^a^	*P* value
**Time point (Baseline)**							
	1 month	1.00	0.55	−0.07 to 2.08	3.32	1	.07
	3 months	0.64	0.65	−0.64 to 1.9	0.92	1	.33
	6 months	0.68	0.74	−0.76 to 2.12	0.86	1	.35
**Caregiver gender (versus female)**							
	Male	3.99	1.20	1.63 to 6.36	11.02	1	.001^b^
**Financial needs met (versus no)**							
	Yes	1.06	1.02	−0.94 to 3.05	1.08	1	.30
Caregiver age		0.14	0.07	0.00 to 0.29	3.88	1	.049^b^
**Study group (versus control)**							
	Treatment	−0.32	0.97	−2.22 to 1.58	0.11	1	.74
**Relationship to care recipient (versus other)**							
	Spouse	−1.58	1.53	−4.59 to 1.42	1.07	1	.30
Years in caregiver role		−0.07	0.13	−0.33 to 0.19	0.28	1	.60
GSES^c^ total score		0.54	0.10	0.34 to 0.741	28.26	1	<.001^a^
SF-12v2 PCS^d^		−0.26	0.04	−0.35 to −0.174	35.37	1	<.001^a^
HHI total score		0.69	0.09	0.52 to 0.870	60.09	1	<.001^a^

^a^*df*: degrees of freedom.

^b^Significant at *P* ≤.05.

^c^GSES: General Self-Efficacy Scale.

^d^SF-12v2 PCS: Short Form-12 item (version 2) physical component summary score.

GEE indicated no statistically significant differences between groups over time in MCS scores (Table 3). Statistically significant variables in the MCS model were male sex (*P*=.001); older age (≥65 years; *P*=.049); and HHI (*P*<.001), GSES *P*<.001) and SF-12v2 PCS (*P*<.001) scores.

**Figure 3:**
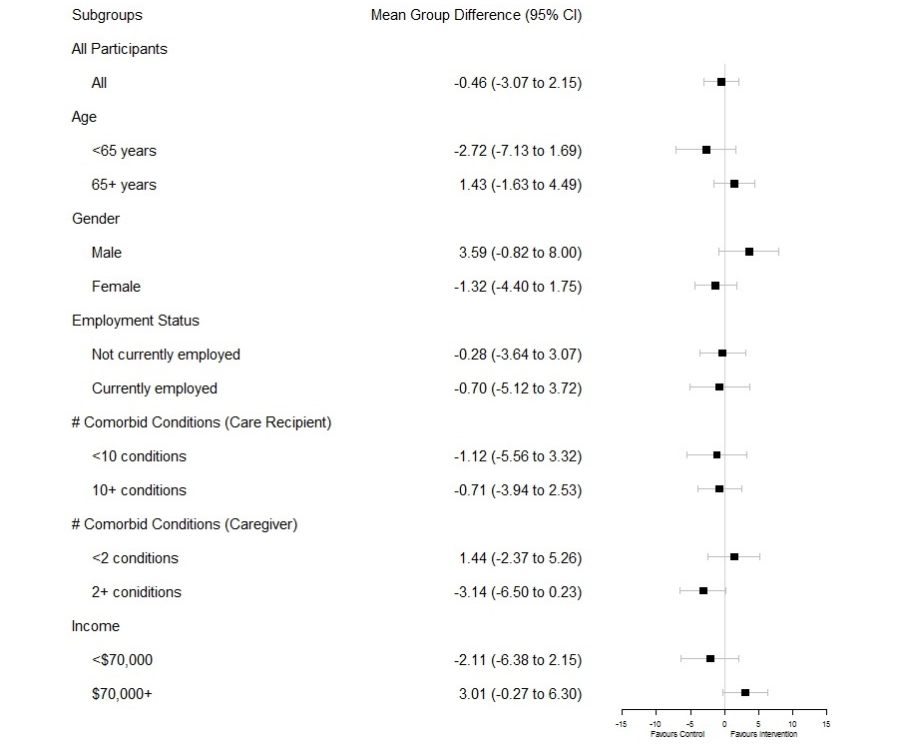
Subanalysis.

Subgroup analysis results also indicated that there were no statistically significant findings for differences in MCS scores (Figure 3).

Although no statistically significant differences between the groups were observed in the primary and secondary outcomes or the subgroup analyses, participants in the treatment group indicated, when asked in interviews, that MT4C helped them with their transitions. For example, one participant said:

“Yeah, it does, because—but it’s how I—you know, I really wish I would have written those things down on the day that they had happened, you know, because it would give me something concrete to go back and see how the—how he digressed...”

Participants who felt MT4C did not help them with transitions suggested it was because they were already privy to sufficient resources. For example, one participant noted that MT4C “didn’t help me significantly...I had gone to some carers’ group and got some information there.”

### Health and Social Service Costs

Table 4 provides the results of comparing the two groups in terms of the change in costs from baseline to 3 months. No statistically significant between-group differences were observed in the cost of individual health and social services or in overall service costs.

**Table 4:** Cost comparison: intervention versus control group (baseline vs 3 months).

Health and social services utilization inventory	Intervention (Can $)				Control (Can $)				Independent samples difference, Wilcoxon Mann–Whitney *z*-statistic (*P* value)^a^
	Baseline median (Q1, Q3)	3-month median (Q1, Q3)	Median cost difference (Q1, Q3)	Baseline median (Q1, Q3)		3-month median (Q1, Q3)	Median cost difference (Q1, Q3)		
Physicians (primary care & specialists)	185.00 (129.94, 340.89)	185.00 (132.95, 370.00)	0.00 (−77.20, 73.48)	185.00 (148.85, 281.71)		185.00 (77.20, 354.88)	0.00 (−123.33, 157.25)	−0.41 (.68)	
Hospital & emergency department	523.02 (283.02, 703.54)	386.04 (283.02, 772.08)	−1726.06 (−3452.12, 0.00)	386.04 (283.02, 1158.12)		386.04 (386.04, 386.04)	772.08 (772.08, 772.08)	0.61 (.54)	
Other health & social service providers	180.00 (87.86, 416,64)	242.87 (102.93, 455.90)	0.00 (−131.20, 131.79)	293.00 (94.87, 690.19)		220.69 (470.52, 111.05)	0.00 (−75.69, 150.96)	−0.03 (.98)	
Laboratory services	24.51 (12.40, 109.44)	41.94 (6.74, 240.50)	−5.58 (−70.00, 25.05)	38.32 (16.45, 145.64)		19.15 (3.29, 114.63)	0.00 (−35.24, 18.18)	−0.93 (.35)	
Prescription medications	80.77 (49.38, 243.82)	240.36 (130.54, 558.34)	−24.60 (−59.72, −10.38)	106.38 (47.43, 227.50)		78.08 (31.55, 134.64)	0.00 (0.00, 0.00)	−1.20 (.23)	
Community support services	165.00 (91.43, 344.93)	206.15 (90.45, 346.25)	0.00 (−115.00, 93.66)	183.24 (121.38, 335.94)		178.50 (67.02, 483.35)	18.00 (−125.84, 167.50)	−0.37 (.71)	
Other services	227.70 (109.98, 609.15)	203.05 (51.26, 450.49)	0.00 (−290.00, 189.98)	201.53 (60.00, 378.00)		226.53 (51.26, 406.10)	3.86 (−95.19, 116.42)	−0.23 (.82)	
Total costs	751.20 (316.55, 1285.13)	587.35 (347.51, 1064.31)	32.21 (−319.38, 412.69)	640.92 (354.96, 1091.31)		659.51 (267.62, 1077.79)	129.16 (−380.16, 394.69)	−0.24 (.81)	

^a^The hypothesis tested was there would be no group differences in median scores baseline-3 month.

## Discussion

### Principal Findings

The purpose of this study was to examine the influence of using a self-administered, multicomponent, Web-based intervention (MT4C) in increasing hope, general self-efficacy, and mental health among carers of community-living persons with ADRD and MCC. Despite there being no significant differences between the treatment (MT4C) and educational control groups in the primary or secondary outcome measures, the treatment group had significantly higher factor 2 hope scores than the control group at 3 months. Factor 2 on HHI is a subscale entitled “positive readiness and expectancy” and reflects the confidence of people in their ability to have a positive future [34]. Statements in this subscale include feelings that “there is a light at the end of the tunnel” and “I have a direction.” This increase in hope is consistent with our adapted transition theory, which suggests that when carers are able to redefine what they perceive as normal, they report increases in hope.

The findings also suggest that hope and general self-efficacy continue to be important variables influencing mental health among carers of community-living older persons with ADRD and MCC. MCC adds additional stress and complexity to the caregiving experience [4], and hope and general self-efficacy have been found to influence the carer’s HRQOL in studies of carers with dementia [5]. As hope and general self-efficacy are significant variables influencing mental health, this finding suggests that the model for the intervention has promise and that activities within MT4C targeted at increasing hope and general self-efficacy should be strengthened. For example, the current activity focused on hope is entitled “Everyday hope” in which participants are asked to consider what would give them hope that day. To strengthen this activity, participants could be encouraged to also view a 15-min film entitled “Connecting with Hope” in which carers of persons with ARDR describe how they maintain hope. An activity focused on self-efficacy currently includes participants identifying their own inner strength. This activity could be strengthened by having participants identify what went well each day, to focus on the positive aspects of caregiving.

As a tailored intervention, instructions for use of MT4C suggest that participants use whatever sections they want, for as long as they want. If the treatment effect for MT4C is reliant upon increasing hope and general self-efficacy, a treatment effect might have been realized if the instructions required participants to focus on the activities specifically designed to increase hope and general self-efficacy in this population. Tailored interventions that consist of multiple components are complex. Moreover, pragmatic trials typically do not focus on mechanisms of action, but instead simply ask whether the intervention worked. For these reasons, we did not focus on determining which component and how much of a component is needed to achieve a treatment effect [45]. Although we tried to capture how much and what sections of MT4C were used through self-report, a significant amount of data was missing. Future research should incorporate the measurement of time spent on individual components into the Web design.

Other Web-based interventions for family carers that reported statistically significant findings focused on outcomes such as anxiety, distress, and depression [1]. Other aspects of mental health and HRQOL might have been more sensitive to a treatment effect as a result of using MT4C. A review of multicomponent interventions for family carers of people with dementia suggests that changing HRQOL through interventions is difficult because HRQOL can quickly deteriorate in carers of persons with dementia [46]. Future evaluation of MT4C should target more specific outcomes, such as anxiety, distress, and depression.

The qualitative data suggested that MT4C helped some participants with the significant changes they experienced as carers. Those who did not feel that MT4C helped them indicated that it was because they were already receiving support from an Alzheimer’s Society. As the majority of the participants were recruited from Alzheimer’s Societies, this could have influenced the outcome of the study.

### Limitations

This study has several limitations. Attrition over time resulted in a study cohort consisting of 166 participants at 3 months (post intervention), which is below the sample size required to determine significance. Although multiple strategies were used, similar to another research, recruiting carers for research was difficult [47]. In addition, since this was a convenience sample, the generalizability of our findings is limited. Future research on MT4C should be conducted with larger sample sizes and should include a more random sampling approach. The participants were well-educated and had access to computers; however, 27% of the treatment group did not use MT4C during the 3-month period. Nonuse of Web-based interventions has been reported in another study [48]. Another limitation is that the majority of participants were recruited from Alzheimer’s Societies and already had access to resources. There is an additional possibility, even with blinding of the consents, that there was contamination as the treatment group participants may have discussed MT4C with the control group participants. Future research should examine whether users of MT4C who are unable to attend Alzheimer’s Society support groups can achieve significant improvements in their mental health. Finally, the limitation regarding participant use of MT4C is of concern. In future research, participants should be instructed more clearly regarding the importance of utilizing MT4C and potentially specific components within; moreover, keeping a track of the time spent on each component can be accomplished through website design strategies.

### Conclusion

This study was unique because MT4C is focused on supporting carers of community-living older persons with ADRD and MCC. Furthermore, it is one of very few studies to include costs from a societal perspective. The findings contribute to future research designs for Web-based interventions with carers as well as future research with MT4C.

## Appendix

Multimedia Appendix 1 Letter of information and consent forms.

Multimedia Appendix 2 Screenshots MT4C.

Multimedia Appendix 3 CONSORT-EHEALTH checklist (V 1.6.1.).

## Abbreviations

ADRD: Alzheimer’s disease and related dementias
ANCOVA: analysis of covariance
GEE: general estimating equations
GSES: General Self-Efficacy Scale
HHI: Herth Hope Index
HRQOL: health-related quality of life
HSSUI: Health and Social Services Utilization Inventory
MCC: multiple chronic conditions
MCS: mental component score SF-12
MT4C: My Tools 4 Care
PCS: physical component score SF-12
QOL: quality of life
RCT: randomized controlled trial
SF-12: Short Form-12 item health survey
