# Supplementary material for: Web-Based Intervention for Family Carers of Persons with Dementia and Multiple Chronic Conditions (My Tools 4 Care): Pragmatic Randomized Controlled Trial
Source: J Med Internet Res. 2018 Jun 29;20(6):e10484. doi: 10.2196/10484 (PMC6045794; doi:10.2196/10484)
Supplement: Multimedia Appendix 1 [file jmir_v20i6e10484_app1.pdf]

UNIVERSITY OF ALBERTA

FACULTY OF NURSING

Wendy Duggleby, *PhD, RN, AOCN*
Professor, Nursing Research Chair Aging and Quality of Life

Level 3, ECHA 11405 – 87 Ave
Edmonton, AB, Canada T6G 1C9
Tel: 780.492.8660 | Fax: 780.492.2551
wendy.duggleby@ualberta.ca

Letter of Information and Consent Form for Family Caregiver Participants Group 1

Study Title: Evaluation of a Transition Intervention for Family Caregivers of Persons with Alzheimer Disease/Related Dementias and Multiple Chronic Conditions

Principal Investigators:

Dr. Wendy Duggleby
Faculty of Nursing
University of Alberta
Edmonton, Alberta
1-877- 692-5909 (toll free)
livingwithhope@nurs.ualberta.ca

Dr. Jenny Ploeg
905-525-9140 (ext. 22294)

Dr. Carrie McAiney
905-521-2100 (ext. 74665)

Research Sponsor: Canadian Institutes of Health Research (CIHR)

Background:

You are being asked to take part in this study because you have a family member or friend who has Alzheimer Disease and a chronic condition. This Information & Consent Form is part of the process of informed consent. It explains this research study and what will happen to you if you choose to be in this study. If you would like to know more about anything you read here, or have any questions at any time regarding this research study, please be sure to ask the researchers or research assistant. Read this form carefully to make sure you understand all the information provided. You will get a copy of this form to keep. You do not have to take part in this study if you don't want to. Your family member's care does not depend on whether or not you take part.

Purpose of the Study:

The purpose of this study is to help us evaluate a new way of supporting family caregivers. We want to know how effective the approach is, and how it may influence factors such as your hope, quality of life, and ability to deal with adverse situations. The overall goal of this research is to help caregivers like you.

Wendy Duggleby, *PhD, RN, AOCN*
Professor, Nursing Research Chair Aging and Quality of Life
Level 3, ECHA 11405 – 87 Ave
Edmonton, AB, Canada T6G 1C9
Tel: 780.492.8660 | Fax: 780.492.2551
wendy.duggleby@ualberta.ca

Procedure:
Approximately 180 people like you will take part in this study, 90 in Alberta and another 90 from Ontario. If you agree to take part in this study, you will be asked to talk with a researcher by telephone for about an hour to complete surveys at four time points (first survey, then 1 month, 3 months and 6 months later). The researcher will ask questions about your health, if you feel you are supported, and if you feel you are able to solve problems. All interviews will be audio-taped.

If you take part in the study, you will be randomly assigned to one of two different ways of supporting family caregivers. Both ways will involve receiving a copy of an educational booklet from the Alzheimer Society.

Note that there is an equal chance of being assigned to either group. You will not know to which group you have been assigned. Knowing more about the two groups may affect the results of the study. At the end of the study, you will be given a letter with more information about the two groups, and be given the opportunity to ask questions.

You will be given instructions on how to access an online toolkit (My Tools 4 Care) for you to use as you wish for 3 months. The information that you add to this online toolkit will be confidential. Only the study administrator will be able to see it in case you need assistance with the website.

In recognition of your time for this study, you will be given a $20.00 coffee gift card. Even though you are receiving this gift, you do not need to answer any questions you don’t want to.

Below is a table of the timing of the surveys that you will be asked to complete:

	**Initial**	**1 month**	**3 months**	**6 months**
Demographic (5min)	X			
Hope (5min)	X	X	X	X
Quality of Life (5 min)	X	X	X	X
Self-Efficacy (5 min)	X	X	X	X
My Tools 4 Care	X	X	x	
Educational Booklet	X			
Interview (15-30 minutes)		X	X	X
Toolkit Checklist (10 min)		X	X	
Use of Services (5 min)	X		X	X

Wendy Duggleby, *PhD, RN, AOCN*
Professor, Nursing Research Chair Aging and Quality of Life
Level 3, ECHA 11405 – 87 Ave
Edmonton, AB, Canada T6G 1C9
Tel: 780.492.8660 | Fax: 780.492.2551
wendy.duggleby@ualberta.ca

Potential Risks:

There are minimal risks to taking part in this study. It is possible you may feel distressed or upset during the interview because talking about your experiences may make you feel sad. You are free to stop an interview or stop taking part in the study at any time, just notify the research assistant. If you want, the research assistant may offer to refer you to someone who is not directly involved with the study, for you to talk to.

There is no cost to you associated with taking part in the study other than your time which will be at the most 4 hours over a 6 month time period, plus time spent on My Tools 4 Care.

Potential Benefits:

The research may benefit you directly, as using My Tools 4 Care may increase your hope. You may also find it helpful to talk about your experience. The results of the research will help us find out the best way to help caregivers like you.

Confidentiality:

Every effort will be made to keep confidential any information that is obtained during this research study. The information collected will not have your name on it. All reporting will be done in a group format, so you will not be able to be identified. Anything that we find out about you that could identify you will not be published or told to anyone. Your identity will remain protected in any publications or presentations of the study results.

What you tell us will be stored in a locked filing cabinet and in a secure place on a computer. Only the research team will see the data and that will not have your name on it.

The data will be kept in a locked cabinet of the nominated Principal Investigator (Wendy Duggleby) for a period of five years following the completion of the study. The findings will be published in scholarly journals as well as presented at various conferences related to caregiving.

Participation and Withdrawal:

Your participation in this study is voluntary. If you decide to take part in the study, you are free to stop at any time, even after signing the consent form or part-way through the study. If you decide to stop taking part in the study, there will be no penalty to yourself. Just let the researcher know. If you do not want to answer some of the questions you do not have to but you can still be in the study. If you would like your data to be destroyed, just let us know.

You may be contacted at a later date for additional opportunities to participate in related research. If contacted, you will be given information about the study and can decide if you would like to participate.

Wendy Duggleby, *PhD, RN, AOCN*
Professor, Nursing Research Chair Aging and Quality of Life
Level 3, ECHA 11405 – 87 Ave
Edmonton, AB, Canada T6G 1C9
Tel: 780.492.8660 | Fax: 780.492.2551
wendy.duggleby@ualberta.ca

Study Debriefing:

You may obtain information about the results of the study by emailing Dr. Wendy Duggleby at: **livingwithhope@nurs.ualberta.ca**. The research findings will be sent via e-mail or by mail to the address you provide.

Rights of Research Participants:

You may withdraw your consent at any time during the study without consequence. If you have any questions or concerns about the research study, please feel free to contact Wendy Duggleby at 1-877-692-5909 (toll free) or by email at **livingwithhope@nurs.ualberta.ca**.

This study has been reviewed and received ethics clearance through the University of Alberta Research Ethics Board. If you have any questions or concerns regarding your rights as a participant, or how this study is being conducted, you may contact the University of Alberta's Research Ethics Office at 780-492-2615. This office has no affiliation with the study investigators.

CONTACT NAMES AND TELEPHONE NUMBERS:

Please contact the individual identified below, at the time of consent and at any time during the study, if you have any questions or concerns about the research project and procedures:

Principal Investigator: Dr. Wendy Duggleby
Faculty of Nursing, University of Alberta
livingwithhope@nurs.ualberta.ca
1-877-692-5909 (toll free)

FACULTY OF NURSING

Wendy Duggleby, *PhD, RN, AOCN*
Professor, Nursing Research Chair Aging and Quality of Life

Level 3, ECHA 11405 – 87 Ave
Edmonton, AB, Canada T6G 1C9
Tel: 780.492.8660 | Fax: 780.492.2551
wendy.duggleby@ualberta.ca

CONSENT FORM

Study Title: Evaluation of a Transition Intervention for Family Caregivers of Persons with Alzheimer Disease/Related Dementias and Multiple Chronic Conditions

Principal Investigator: Dr. Wendy Duggleby
Faculty of Nursing, University of Alberta
Edmonton, Alberta, Canada
1-877- 692-5909 (toll free)
livingwithhope@nurs.ualberta.ca

	**Yes**	**No**
Do you understand that you have been asked to be in a research study?	☐	☐
Have you read and received a copy of the attached Information Sheet?	☐	☐
Do you understand the benefits and risks involved in taking part in this research study?	☐	☐
Have you had an opportunity to ask questions and discuss this study?	☐	☐
Do you understand that you are free to leave the study at any time, without having to give a reason and without affecting your loved one's medical care?	☐	☐
Has the issue of confidentiality been explained to you?	☐	☐
Do you understand who will have access to your data, including personally identifiable information?	☐	☐
Who explained this study to you? ________________________________		
I agree to take part in this study.	☐	☐

Oral consent of research participant:

________________________________ ________________________________
Name of Participant Date

Signature of Person Obtaining Consent: I believe that the participant understands what is involved in the study and voluntarily agrees to take part in.

________________________________ ________________________________ ______________
Name of Person Obtaining Consent (please print) Signature of Person Obtaining Consent Date

UNIVERSITY OF ALBERTA

FACULTY OF NURSING

Wendy Duggleby, *PhD, RN, AOCN*
Professor, Nursing Research Chair Aging and Quality of Life
Level 3, ECHA 11405 – 87 Ave
Edmonton, AB, Canada T6G 1C9
Tel: 780.492.8660 | Fax: 780.492.2551
wendy.duggleby@ualberta.ca

Letter of Information and Consent Form for Family Caregiver Participants: Group 2

**Study Title:**	Evaluation of a Transition Intervention for Family Caregivers of Persons with Alzheimer Disease/Related Dementias and Multiple Chronic Conditions
**Principal Investigators:**	Dr. Wendy Duggleby<br>Faculty of Nursing<br>University of Alberta<br>Edmonton, Alberta<br>1-877- 692-5909 (toll free)<br>**livingwithhope@nurs.ualberta.ca**<br><br>Dr. Jenny Ploeg<br>905-525-9140 (ext. 22294)<br><br>Dr. Carrie McAiney<br>905-521-2100 (ext. 74665)
**Research Sponsor:**	Canadian Institutes of Health Research (CIHR)

Background:

You are being asked to take part in this study because you have a family member or friend who has Alzheimer Disease and a chronic condition. This Information & Consent Form is part of the process of informed consent. It explains this research study and what will happen to you if you choose to be in this study. If you would like to know more about anything you read here, or have any questions at any time regarding this research study, please be sure to ask the researchers or research assistant. Read this form carefully to make sure you understand all the information provided. You will get a copy of this form to keep. You do not have to take part in this study if you don't want to. Your family member's care does not depend on whether or not you take part.

Purpose of the Study:

The purpose of this study is to help us evaluate a new way of supporting family caregivers. We want to know how effective the approach is, and how it may influence factors such as your hope, quality of life, and ability to deal with adverse situations. The overall goal of this research is to help caregivers like you.

UNIVERSITY OF ALBERTA

FACULTY OF NURSING

Wendy Duggleby, *PhD, RN, AOCN*
Professor, Nursing Research Chair Aging and Quality of Life
Level 3, ECHA 11405 – 87 Ave
Edmonton, AB, Canada T6G 1C9
Tel: 780.492.8660 | Fax: 780.492.2551
wendy.duggleby@ualberta.ca

Procedure:

Approximately 180 people like you will take part in this study, 90 in Alberta and another 90 from Ontario. If you agree to take part in this study, you will be asked to talk with a researcher by telephone for about an hour to complete surveys at four time points (first survey, then 1 month, 3 months and 6 months later). The researcher will ask questions about your health, if you feel you are supported, and if you feel you are able to solve problems. All interviews will be audio-taped.

If you take part in the study, you will be randomly assigned to one of two different ways of supporting family caregivers. Both ways will involve receiving a copy of an educational booklet from the Alzheimer Society.

Note that there is an equal chance of being assigned to either group. You will not know to which group you have been assigned. Knowing more about the two groups may affect the results of the study. At the end of the study, you will be given a letter with more information about the two groups, and be given the opportunity to receive the approach that the other group had, and to ask any questions.

In recognition of your time for this study, you will be given a $20.00 coffee gift card. Even though you are receiving this gift, you do not need to answer any questions you don't want to.

Below is a table of the timing of the surveys that you will be asked to complete:

	**Initial**	**1 month**	**3 months**	**6 months**
Demographic (5min)	X			
Hope (5min)	X	X	X	X
Quality of Life (5 min)	X	X	X	X
Self-Efficacy (5 min)	X	X	X	X
Educational Booklet	X			
Interview (15-30 minutes)			X	X
Use of Services (5 min)	X		X	X

Potential Risks:

There are minimal risks to taking part in this study. It is possible you may feel distressed or upset during the interview because talking about your experiences may make you feel sad.

Wendy Duggleby, *PhD, RN, AOCN*
Professor, Nursing Research Chair Aging and Quality of Life

Level 3, ECHA 11405 – 87 Ave
Edmonton, AB, Canada T6G 1C9
Tel: 780.492.8660 | Fax: 780.492.2551
wendy.duggleby@ualberta.ca

You are free to stop an interview or stop taking part in the study at any time, just notify the research assistant. If you want, the research assistant may offer to refer you to someone who is not directly involved with the study, for you to talk to.

There is no cost to you associated with taking part in the study other than your time which will be at the most 4 hours over a 6 month time period.

Potential Benefits:

The research may benefit you directly, as your participation may increase your hope. You may also find it helpful to talk about your experience. The results of the research will help us find out the best way to help caregivers like you.

Confidentiality:

Every effort will be made to keep confidential any information that is obtained during this research study. The information collected will not have your name on it. All reporting will be done in a group format, so you will not be able to be identified. Anything that we find out about you that could identify you will not be published or told to anyone. Your identity will remain protected in any publications or presentations of the study results.

What you tell us will be stored in a locked filing cabinet and in a secure place on a computer. Only the research team will see the data and that will not have your name on it.

The data will be kept in a locked cabinet of the nominated Principal Investigator (Wendy Duggleby) for a period of five years following the completion of the study. The findings will be published in scholarly journals as well as presented at various conferences related to caregiving.

Participation and Withdrawal:

Your participation in this study is voluntary. If you decide to take part in the study, you are free to stop at any time, even after signing the consent form or part-way through the study. If you decide to stop taking part in the study, there will be no penalty to yourself. Just let the researcher know. If you do not want to answer some of the questions you do not have to but you can still be in the study. If you would like your data to be destroyed, just let us know.

You may be contacted at a later date for additional opportunities to participate in related research. If contacted, you will be given information about the study and can decide if you would like to participate.

Wendy Duggleby, *PhD, RN, AOCN*
Professor, Nursing Research Chair Aging and Quality of Life

Level 3, ECHA 11405 – 87 Ave
Edmonton, AB, Canada T6G 1C9
Tel: 780.492.8660 | Fax: 780.492.2551
wendy.duggleby@ualberta.ca

Study Debriefing:

You may obtain information about the results of the study by emailing Dr. Wendy Duggleby at: **livingwithhope@nurs.ualberta.ca**. The research findings will be sent via e-mail or by mail to the address you provide.

Rights of Research Participants:

You may withdraw your consent at any time during the study without consequence. If you have any questions or concerns about the research study, please feel free to contact Wendy Duggleby at 1-877-692-5909 (toll free) or by email at **livingwithhope@nurs.ualberta.ca**.

This study has been reviewed and received ethics clearance through the University of Alberta Research Ethics Board. If you have any questions or concerns regarding your rights as a participant, or how this study is being conducted, you may contact the University of Alberta's Research Ethics Office at 780-492-2615. This office has no affiliation with the study investigators.

CONTACT NAMES AND TELEPHONE NUMBERS:

Please contact the individual identified below, at the time of consent and at any time during the study, if you have any questions or concerns about the research project and procedures:

Principal Investigator: Dr. Wendy Duggleby
Faculty of Nursing, University of Alberta
livingwithhope@nurs.ualberta.ca
1-877-692-5909 (toll free)

Wendy Duggleby, *PhD, RN, AOCN*
Professor, Nursing Research Chair Aging and Quality of Life
Level 3, ECHA 11405 – 87 Ave
Edmonton, AB, Canada T6G 1C9
Tel: 780.492.8660 | Fax: 780.492.2551
wendy.duggleby@ualberta.ca

CONSENT FORM

Study Title: Evaluation of a Transition Intervention for Family Caregivers of Persons with Alzheimer Disease/Related Dementias and Multiple Chronic Conditions

Principal Investigator: Dr. Wendy Duggleby
Faculty of Nursing, University of Alberta
Edmonton, Alberta, Canada
1-877- 692-5909 (toll free)
livingwithhope@nurs.ualberta.ca

	**Yes**	**No**
Do you understand that you have been asked to be in a research study?	☐	☐
Have you read and received a copy of the attached Information Sheet?	☐	☐
Do you understand the benefits and risks involved in taking part in this research study?	☐	☐
Have you had an opportunity to ask questions and discuss this study?	☐	☐
Do you understand that you are free to leave the study at any time, without having to give a reason and without affecting your loved one's medical care?	☐	☐
Has the issue of confidentiality been explained to you?	☐	☐
Do you understand who will have access to your data, including personally identifiable information?	☐	☐
Who explained this study to you? ______________________________		
I agree to take part in this study.	☐	☐

Oral consent of research participant:

______________________________ ______________________________
Name of Participant Date

Signature of Person Obtaining Consent: I believe that the participant understands what is involved in the study and voluntarily agrees to take part in.

______________________________ ______________________________ ________________
Name of Person Obtaining Consent (please print) Signature of Person Obtaining Consent Date
