# Supplementary material for: Web-Based Intervention for Family Carers of Persons with Dementia and Multiple Chronic Conditions (My Tools 4 Care): Pragmatic Randomized Controlled Trial
Source: J Med Internet Res. 2018 Jun 29;20(6):e10484. doi: 10.2196/10484 (PMC6045794; doi:10.2196/10484)
Supplement: Multimedia Appendix 2 [file jmir_v20i6e10484_app2.pdf]

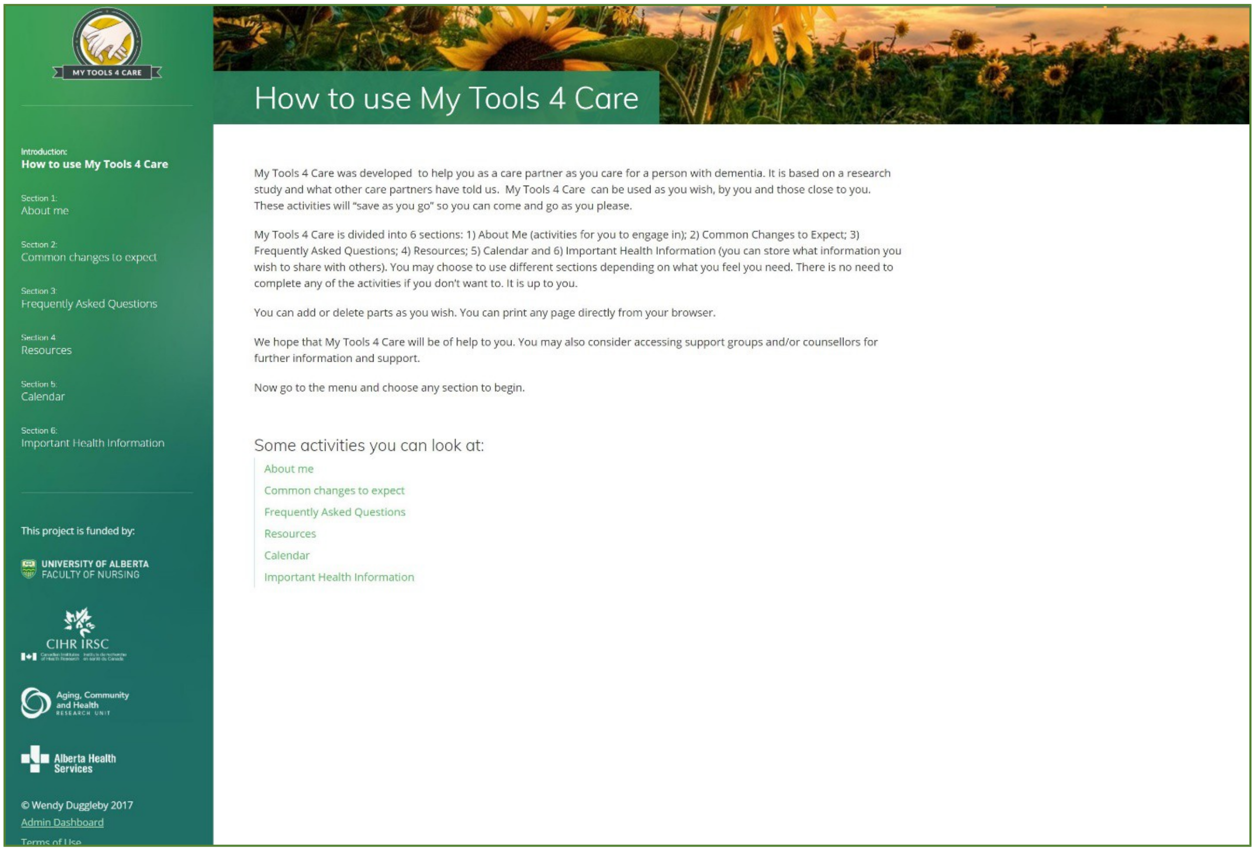
MY TOOLS 4 CARE
Introduction:
How to use My Tools 4 Care
Section 1:
About me
Section 2:
Common changes to expect
Section 3:
Frequently Asked Questions
Section 4:
Resources
Section 5:
Calendar
Section 6:
Important Health Information
This project is funded by:
UNIVERSITY OF ALBERTA FACULTY OF NURSING
CIHR IRSC
Aging, Community and Health RESEARCH UNIT
Alberta Health Services
© Wendy Duggleby 2017
Admin Dashboard
Terms of Use
How to use My Tools 4 Care
My Tools 4 Care was developed to help you as a care partner as you care for a person with dementia. It is based on a research study and what other care partners have told us. My Tools 4 Care can be used as you wish, by you and those close to you. These activities will "save as you go" so you can come and go as you please.
My Tools 4 Care is divided into 6 sections: 1) About Me (activities for you to engage in); 2) Common Changes to Expect; 3) Frequently Asked Questions; 4) Resources; 5) Calendar and 6) Important Health Information (you can store what information you wish to share with others). You may choose to use different sections depending on what you feel you need. There is no need to complete any of the activities if you don't want to. It is up to you.
You can add or delete parts as you wish. You can print any page directly from your browser.
We hope that My Tools 4 Care will be of help to you. You may also consider accessing support groups and/or counsellors for further information and support.
Now go to the menu and choose any section to begin.
Some activities you can look at:
About me
Common changes to expect
Frequently Asked Questions
Resources
Calendar
Important Health Information

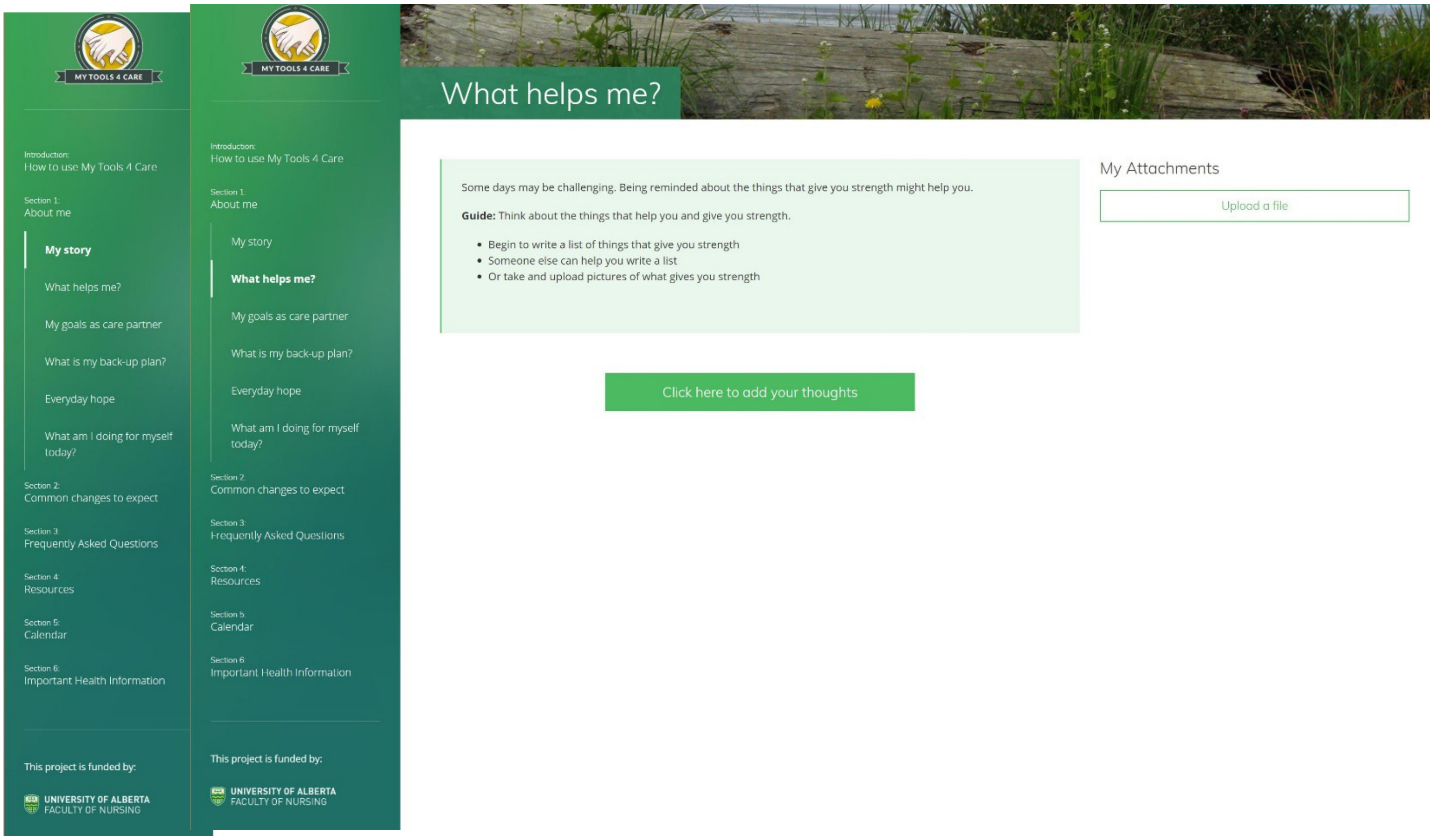
MY TOOLS 4 CARE
Introduction:
How to use My Tools 4 Care
Section 1:
About me
My story
What helps me?
My goals as care partner
What is my back-up plan?
Everyday hope
What am I doing for myself today?
Section 2:
Common changes to expect
Section 3:
Frequently Asked Questions
Section 4:
Resources
Section 5:
Calendar
Section 6:
Important Health Information
This project is funded by:
UNIVERSITY OF ALBERTA FACULTY OF NURSING
What helps me?
Some days may be challenging. Being reminded about the things that give you strength might help you.
Guide: Think about the things that help you and give you strength.
Begin to write a list of things that give you strength
Someone else can help you write a list
Or take and upload pictures of what gives you strength
Click here to add your thoughts
My Attachments
Upload a file

MY TOOLS 4 CARE

Introduction:
How to use My Tools 4 Care

Section 1:
About me

My story

What helps me?

My goals as care partner

What is my back-up plan?

Everyday hope

What am I doing for myself today?

Section 2:
Common changes to expect

Section 3:
Frequently Asked Questions

Section 4:
Resources

Section 5:
Calendar

Section 6:
Important Health Information

This project is funded by:

UNIVERSITY OF ALBERTA
FACULTY OF NURSING

UNIVERSITY OF ALBERTA
FACULTY OF NURSING

MY TOOLS 4 CARE

Introduction:
How to use My Tools 4 Care

Section 1:
About me

My story

What helps me?

My goals as care partner

What is my back-up plan?

Everyday hope

What am I doing for myself today?

Section 2:
Common changes to expect

Section 3:
Frequently Asked Questions

Section 4:
Resources

Section 5:
Calendar

Section 6:
Important Health Information

This project is funded by:

UNIVERSITY OF ALBERTA
FACULTY OF NURSING

What am I doing for myself today?

You need to keep yourself healthy and well in order to care for others. It's up to you; nobody else is able to do it for you.

Guide: Do something for yourself to avoid caregiver stress. Decide what you want to do (not what you should do, or have a need to do) and write some ideas below. For example, going for a 20 minute walk, or connecting with other care partners.

- How much will you do?
- When will you do it?
- What resources do you need to do it?
- Don't worry if you can't do it, try again tomorrow

One way of connecting with other care partners is through the ASANT Cafe. This online community is listed in the resource section.

Click here to add your thoughts

My Attachments

Upload a file

MY TOOLS 4 CARE

Introduction:
How to use My Tools 4 Care

Section 1:
About me

My story

What helps me?

My goals as care partner

What is my back-up plan?

Everyday hope

What am I doing for myself today?

Section 2:
Common changes to expect

Section 3:
Frequently Asked Questions

Section 4:
Resources

Section 5:
Calendar

Section 6:
Important Health Information

This project is funded by:

UNIVERSITY OF ALBERTA
FACULTY OF NURSING

Everyday hope

Some people find that writing and then thinking about what gives them hope can be useful to gain or keep a sense of hope.

Guide: Write below what will give you hope today. What helps you to have hope today is an exercise you can do every day.

- Think of what will give you hope or help today
- Think of one big or small thing you would like to finish today
- What helps you to have hope?

Connecting with Hope

Is a video that reflects the hope experience of family caregivers of persons with dementia residing in Long Term Care Facilities. It was funded by the Nursing Research Chair in Aging and Quality of Life (15 minutes).

Use the following link to see the film: www.nurs.ualberta.ca/livingwithhope

Click here to add your thoughts

My Attachments

Upload a file

MY TOOLS 4 CARE

Introduction:
How to use My Tools 4 Care

Section 1:
About me

Section 2:
Common changes to expect

Section 3:
Frequently Asked Questions

Section 4:
Resources

Section 5:
Calendar

Section 6:
Important Health Information

This project is funded by:

UNIVERSITY OF ALBERTA
FACULTY OF NURSING

CIHR IRSC

Aging, Community and Health RESEARCH UNIT

Alberta Health Services

© Wendy Duggleby 2017
Admin Dashboard
Terms of Use

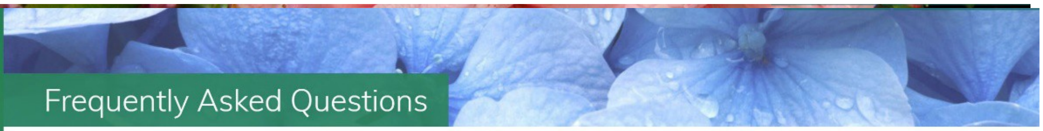

Frequently Asked Questions

Other people providing care to a family member with dementia have asked questions about the changes they experienced. In this section the most common questions are listed with answers provided. We hope these are helpful to you.

You may have other questions that are not listed here. The resources listed in the next section may help you find answers to those questions.

If a service I require is not available, how do I access this service?
Call your local doctor or Alzheimer Society. You can find your local Alzheimer Society by contacting the Alzheimer Society of Canada.

How do I find information about disease and the symptoms I should expect?
Contact your local doctor or Alzheimer Society about getting information and ask first for their suggestions to how you can get information. There are some internet references that we have listed on the resources page for you.

Are there books or websites available that would be of help to me?
See the resources section of this toolkit for a list of useful items.

If I need to talk to a doctor or any other health care professional, what should I do?
If reaching your doctor is difficult, call your local Alzheimer Society for assistance.

What do we do if the doctor doesn't see the changes?
Keep track of what you see and use the behavoiral tracking form under the "Important Health Information" section. Then share the written information with your doctor.

What do I do if the doctor won't talk to me?
Have the person you are caring for write a note, or tell the doctor that they agree that you are to have all the information about them and that they want the doctor to talk with you.

How do I find information on medications?
You should consult locally with the pharmacist first. Pharmacies give information packages on the medication. A good website for information on medications can be found in the resources section.

MY TOOLS 4 CARE

Introduction:
How to use My Tools 4 Care

Section 1:
About me

Section 2:
Common changes to expect

Section 3:
Frequently Asked Questions

Section 4:
Resources

Your contact list

National contacts

Helpful Resources

Planning

Section 5:
Calendar

Section 6:
Important Health Information

This project is funded by:

UNIVERSITY OF ALBERTA
FACULTY OF NURSING

CIHR IRSC

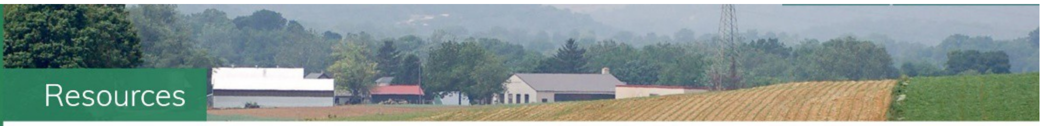

Resources

This section contains information about resources to help you as a care partner prepare for possible changes or events, and to connect you with services in your community. This section also includes a contact list template for you to fill in should you desire to do so. This section is based on what other care partners have told us is helpful to them.

Some activities you can look at:

- Your contact list
- National contacts
- Helpful Resources
- Planning
